# Uncertainty in illness in family caregivers of palliative care patients and associated factors

**DOI:** 10.1590/1518-8345.3185.3200

**Published:** 2019-10-14

**Authors:** Mauricio Arias-Rojas, Sonia Carreño-Moreno, Carolina Posada-López

**Affiliations:** 1 Universidad Nacional de Colombia, Facultad de Enfermería, Bogotá, Cund, Colômbia.; 2 Universidad de Antioquia, Facultad de Enfermería, Medellín, Ant, Colômbia.

**Keywords:** Uncertainty, Chronic Disease, Caregivers, Family, Nursing Theory, Palliative Care, Incerteza, Doença Crônica, Cuidados Paliativos, Cuidadores, Família, Teoria de Enfermagem, Incertidumbre, Enfermedad Crónica, Cuidadores, Familia, Teoría de Enfermería, Cuidados Paliativos

## Abstract

**Objective:**

to describe the level of uncertainty in illness in family caregivers of palliative care patients and detect associations between the profile of the caregiver and the levels of uncertainty.

**Method:**

descriptive correlational study conducted with 300 family caregivers of hospitalized patients. The sociodemographic characterization of caregiver and patient was used to assess the caregiver profile, as well as the Uncertainty in Illness scale for family caregivers. Spearman’s Rho correlation test was applied to detect associations.

**Results:**

the average score of illness uncertainty was 91.7 points. The analysis showed significant correlations between the level of uncertainty and patient dependence (r=0.18, p=0.001), symptom assessment (r=0.312, p<0.001), length of service as a caregiver (r=0.131, p=0.023), perception of support from health professionals (r=-0.16, p=0.048), family (r=-0.145, p=0.012) and religious support (r=-0.131, p=0.050).

**Conclusions:**

there were high levels of uncertainty in caregivers about their patient’s illness. These levels are associated with the health condition and symptoms of the patient who is cared for, the length of service as a caregiver and the perceived support from health professionals, family and religion.

## Abstract (pt)

**Objetivo:**

descrever o nível de incerteza dos cuidadores familiares na doença de pacientes sob cuidados paliativos e descobrir associações entre o perfil do cuidador e os níveis de incerteza.

**Método:**

estudo correlacional descritivo, realizado com 300 cuidadores familiares de pacientes hospitalizados. A caracterização sociodemográfica do cuidador e do paciente foram utilizadas para avaliar o perfil do cuidador, assim como a escala Incerteza na Doença do cuidador familiar. O teste de correlação Rô de Spearman foi aplicado para detectar associações.

**Resultados:**

a média encontrada para o nível de incerteza da doença foi de 91,7 pontos. As análises revelaram correlações significativas entre o nível de incerteza e a dependência do paciente (r=0,18; p=0,001), avaliação dos sintomas (r=0,312; p<0,001) tempo como cuidador (r=0,131; p=0,023), percepção de apoio dos profissionais de saúde (r=-0,16; p=0,048), família (r=-0,145; p=0,012) e apoio religioso (r=-0,131; p=0,050).

**Conclusões:**

há altos níveis de incerteza do cuidador na doença do paciente. Esses níveis estão associados com a condição de saúde e os sintomas do paciente cuidado, o tempo como cuidador e o apoio percebido dos profissionais de saúde, família e religião.

## Abstract (es)

**Objetivo:**

describir el nivel de incertidumbre de cuidadores familiares ante la enfermedad de pacientes en cuidados paliativos y explorar asociaciones entre el perfil del cuidador y los niveles de incertidumbre.

**Método:**

descriptivo correlacional, realizado con 300 cuidadores familiares de pacientes hospitalizados. Se utilizó para medir el perfil del cuidador una caracterización sociodemográfica de este y del paciente, así como la escala de Incertidumbre Ante la Enfermedad del cuidador familiar. Se aplicó la prueba de correlación Rho de Spearman para detectar asociaciones.

**Resultados:**

se encontró un nivel de incertidumbre ante la enfermedad promedio de 91,7 puntos. Los análisis revelaron correlaciones significativas entre el nivel de incertidumbre y la dependencia del paciente (r=0,18; p=0,001), evaluación de síntomas (r=0,312; p<0,001) tiempo como cuidador (r=0,131; p=0,023), percepción de apoyo de los profesionales de la salud (r=-0,16; p=0,048), familia (r=-0,145; p=0,012) y apoyo religioso (r=-0,131; p=0,050).

**Conclusiones:**

existen altos niveles de incertidumbre del cuidador ante la enfermedad del paciente; estos niveles se asocian con la condición y los síntomas del paciente al que se cuida, el tiempo como cuidador y el soporte percibido por parte de profesionales de la salud, la familia y la religión.

## Introduction

Chronic Noncommunicable Diseases (CNCD) are a global public health problem that affect both developed and developing countries^1^. As CNCD and demands for care increase, the need for specialized care, such as palliative care (PC) also increases^2^.

People with CNCD, as their disease progresses and they approach the end of their life, they need to make lifestyle changes and restructure their lives to adjust to new conditions, which generate increasingly dependency and deterioration^3^. For a person to make this adjustment, in many cases it requires the support of a family caregiver, who takes on the role of accompaniment, direct care, health management, among other activities^4^. Often, when assuming this role, there is a lack of knowledge of basic aspects related to the diagnosis of the patients, their type of treatment, their prognosis, as well as the care for the patient^5-6^. Taking on these tasks without the necessary knowledge brings feelings of overload, anxiety, stress, physical problems and decreased quality of life^7^.

In 1988, Merle Mishel theorized that the lack of knowledge of the issues related to the illness leads the person to experience a state of uncertainty, which she described in her theory on Uncertainty in Illness, a theory that can be applied to patients, caregivers and parents of children^8^. For this author, uncertainty in illness refers to the inability to determine the meaning illness related events due to insufficient signals to do so^8^. In other words, uncertainty arises in response to ambiguous illness related events due to insufficient information, unpredictable or changing symptoms, ambiguous disease trajectory, insufficient social support, inadequate support from health professionals and the cognitive abilities to understand the development of the disease^9^.

Regarding the CNCD, some studies have reported that the high levels of illness uncertainty experienced by caregivers are associated with aspects such as decreased ability to learn and understand novel information^10^, inability to predict the results of events presented by the patient, adapt to the diagnosis of the disease and low levels of quality of life^11-12^. Despite the evidence on the phenomenon of uncertainty in CNCD, such as cancer and other pathologies, it is important to understand that the application of this nursing theory in the field of PC provides tools for nursing professionals to understand and help the family caregiver who experiences uncertainty in illness of the patient. In addition, the usefulness of its application can be demonstrated in another context of care, to which it has not yet been used and, thus, new disciplinary knowledge is produced with respect to this theory.

For nursing professionals, recognizing the level of uncertainty of these caregivers and the factors that may be associated with it, allows nursing interventions focused on the aspects that generate caregiver uncertainty, which will result in the improvement of patient care. In view of the above, it is important to describe the level of uncertainty in illness in family caregivers of patients in PC and detect associations between the profile of these family caregivers and the levels of uncertainty they may have.

## Method

This is a descriptive cross-sectional study. This study was conducted in a private hospital of high complexity in Bogotá-Colombia during the second semester of 2017 and the first quarter of 2018.

Participants were selected through a review of patients in PC admitted to the hospital and who were cared for by the PC support team. The sample selection was carried out in an intentional way by one of the study researchers, who reviewed the list of patients hospitalized and cared for by the PC support team and invited those who met the inclusion criteria to participate. The inclusion criteria were: (a) to be the main caregiver of the patient and the person responsible for their care most of the time, (b) to be over 18 years of age, (c) to have unaltered state of consciousness and orientation, (d) patient must have an advanced disease and be cared for by the PC support group. The following exclusion criteria were considered: (a) to be a professional caregiver or a caregiver hired to care for the patient.

Caregiver profile. This profile included the sociodemographic characteristics of the caregiver, the workload and support they have received, the use and management of technologies, and the patient’s condition. The profile was measure by the characterization card of the dyad in PC, an instrument that explores the sociodemographic aspects of the family caregiver of a person in PC and the patient cared for^13^. The card is divided into three sections: the first section explores the sociodemographic profile of the caregiver and includes items such as age, gender, education, occupation, socioeconomic status, among others. For the classification of the socioeconomic status, it was used the Colombian social-economic stratification, according to the National Planning Department. The second section explores aspects related to the perception of workload and support provided to the caregiver, and includes items such as the length of service as a caregiver, the support provided to the caregiver and the level of satisfaction, the level of well-being of the caregiver and the perception of overload caused by patient care. To measure the level of satisfaction with the support provided, the card uses a scale with 4 response options ranging from 1 (low) to 4 (high). To measure the level of well-being, it has 5 response options ranging from 0 (insufficient) to 4 (high). To measure the workload level, it has 4 response options ranging from no overload to severe overload. The third section includes items on the use and management of information and communication technologies (ICTs) for patient care, which is measured by a dichotomous response option (yes/no).

Complementing the caregiver profile, the card also includes measures of the clinical variables of the patient, which are the patient’s functionality and the perception of patient’s symptoms. For this, the Karnofsky Performance Scale index (KPS) was used, which assesses the functional status of a patient, with 11 response options ranging from 0 (dead) to 100 (fully active). Information from the Edmonton Symptom Assessment Questionnaire^14^ (ESAS-R) was also used on the day the caregiver was interviewed to observe the prevalence of symptoms experienced by the patients under their care. This scale includes the 10 main symptoms experienced in palliative care, and has 11 response options ranging from 0 to 10, where zero represents no experience of the symptom and 10 represents the worst experience of this symptom, and this scale does not have a total score and each symptom is observed separately.

To measure the uncertainty, the Uncertainty in Illness Scale for Family Caregivers was used, which was designed to measure the uncertainty experienced by a caregiver about the patient’s illness^15^. This instrument has 31 items with a Likert type response scale that ranges from strongly agree (5 points) to strongly disagree (1 point). The participant’s responses are based on their perception of the current health situation of the patient. This instrument includes statements such as “I have many unanswered questions about my family member’s illness”, “I understand everything the health professional explains about my family member’s illness”, “the effectiveness of the treatment for my family member’s illness is not clear” among others. The instrument scores range from 31 to 155 points and the higher the score, the greater the degree of illness uncertainty. According to the author, a low illness uncertainty score is that below 59 points, medium from 59 to 87 points and high that above 87 points.

The scale shows construct validity in its original version and internal consistency for caregivers of patients with different chronic diseases, and its alpha coefficient can vary from 0.64 to 0.89 in each dimension^15^. In its version in Spanish, the scale was adapted and validated for a study of parent caregivers of children, showing a construct validity with 3 factors and an internal consistency with a Cronbach’s alpha of 0.86^16^. It was also validated for caregivers of people in palliative care, with an internal consistency of 0.79.

The data was analyzed using the SPSS 20.0^®^ statistical program for Windows. The continuous variables were expressed as mean and standard deviation, and the qualitative variables as frequencies. To detect correlations between the variables, the Kolmogorov Smirnov test for normality was used. Pearson’s R correlation test was applied for those variables that met the requirements of normality and Spearman’s Rho test was used for those that did not meet. The statistical significance was set as p < 0.05, and for the interpretation of the correlations obtained the p value was set as: < 0.3 weak magnitude, ≥ 0.3 to < 0.6 moderate magnitude and ≥ 0.6 strong magnitude^17^.

The research ethics committee of the health institution where the study was carried out and the ethics committee of the Faculty of Nursing of the National University of Colombia approved this study, protocol no. Aval-009-17. The participants were informed of the research objectives and voluntarily signed the informed consent form of the study.

## Results

During the study period, 323 family caregivers who met the inclusion criteria were invited to participate in the study. Of these, 300 agreed to participate in the study. The results are shown below.

Regarding the profile of the family caregiver, in terms of sociodemographic characteristics, it was found an average age of 50.17 years (Standard Deviation, SD = 13.75) with a maximum age of 84 years and a minimum age of 18 years. As for the gender, 79% were female, with secondary education in 62% of cases, higher education in 22.67% and primary education in 14.67% of cases. As regards the marital status of the caregivers, 39% were married, 21.33% were in a long-term relationship and 25.33% were single. As for the socioeconomic status, it was found that 45.67% had a medium socioeconomic status, 35.33% low, 12.33% high and only 6.67% very low.

The detailed aspects of the characteristics of the role of the caregiver of a patient in PC are described in Table 1.

**Table 1:** Characterization of the role of the caregiver of a patient in palliative care. Bogotá, Cundinamarca, Colombia, 2017-2018

Variable	Percentage % (n=300)
Care for the person since diagnosis	
Yes	90%
No	10%
Sole caregiver	
Yes	74.67%
No	25.33%
Length of service as a caregiver in years	
Mean	6.7365
Standard Deviation	7.13636
Minimum	0.08
Maximum	34
Number of daily hours of care	
Media	12.383
Standard Deviation	6.654
Minimum	3
Maximum	24
Previous experiences as caregiver	
Yes	57%
No	43%
Level of perception of workload with patient care	
No overload	26.33%
Mild overload	59%
High overload	0.33%
Severe overload	14.33%

The level of well-being and perception of support by the caregiver of a patient in PC are described in Table 2.

**Table 2:** – Level of well-being and perception of support by the family caregivers of palliative care patients. Bogotá, Cundinamarca, Colombia, 2017-2018

Variable	Mean n = 300	Standard Deviation
Level of perceived support from health professionals	3.55	0.723
Level of perceived family support	3.51	0.816
Level of perceived religious support	3.23	0.998
Level of perceived social support	3.49	0.900
Level of physical well-being	2.67	1.128
Level of psychological and emotional well-being	2.76	1.176
Level of social well-being	3.07	0.979
Level of spiritual well-being	3.16	1.083

Use of ICTs^*^ in patient care	**Percentage**	

Yes	56.33%	
No	43.67%	

*ICTs = Information and communication technologies

As regards the patient cared for by the family caregiver, it was found that 54% were female, with an average age of 70.9 years and a standard deviation (SD) of 14.03. Regarding the underlying disease, 43.3% had cardiovascular disease, 25% cancer, 12% metabolic diseases, 9.6% kidney disease, 8.6% lung disease and 1.3% had neurological diseases. In terms of functional status, most of them achieved 40 points (36%) and 50 points (33.3%), followed by 60 points (11.67%) and 30 points (6%). As for the symptoms assessment using the ESAS-R, the following mean scores were found: Pain=2.94; Fatigue=5.56; Sleepiness=2.99; Nausea=1.23; Loss of appetite=3.33; Difficulty breathing=4.49; Discouragement=4.06; Restlessness=3.33; Sleep=4.47; Well-being=4.86.

In relation to the main objective of this study, it was found that family caregivers had an average of illness uncertainty of 91.7 points, with an SD of 8.8 points, a maximum of 124 and a minimum of 66 points. This result reveals a high level of illness uncertainty.

Regarding the possible associations between the profile of the caregiver and the levels of illness uncertainty, no correlation was found between the level of illness uncertainty and some of the characteristics of the caregiver profile when the exploratory analysis of the sociodemographic variables was performed. These characteristics of the caregiver profile are level of education, being the only caregiver, level of perceived social support, previous experiences as caregiver, level of perception of workload with patient care, use of TICs, well-being and socioeconomic status. However, six characteristics in the caregiver profile showed significant weak and moderate correlations with the level of uncertainty in caregivers about the patient’s illness, which are shown below (Table 3).

**Table 3:** Matrix of Spearman’s correlation coefficients for Uncertainty in Illness in family caregivers and the study variables. Bogotá, Cundinamarca, Colombia, 2017-2018

Variables		KPS^*^	ESAS R^†^	Length of service as a caregiver Healthcare professionals	Level of perceived support		

					Family	Religion	
Uncertainty in caregivers about the patient’s illness	Coefficient	0.187^‡^	0.312^‡^	0.131^§^	-0.16^§^	-0.145^§^	-0.113^§^
	Type	Weak	Moderate	Weak	Weak	Weak	Weak
	Value p^║^	0.001	0.000	0.023	0.048	0.012	0.050

*KPS = Karnofsky Performance Scale index; ^†^ESAS-R = Edmonton symptom assessment questionnaire; ^‡^correlation is significant at the 0.01 level (unilateral); ^§^correlation is significant at the 0.05 level (bilateral); ^|^p = statistical significance

## Discussion

This study aimed to describe the uncertainty in illness in caregivers of patients in PC and to detect associations between the profile of these family caregivers and their levels of illness uncertainty.

Regarding the level of uncertainty in caregivers about the patient’s illness, a high level of uncertainty was found in this group of participants. In this regard, the literature reports that the levels of illness uncertainty in caregivers of patients with stroke were medium levels^18^. Another study conducted by the same researchers^19^ found a medium level of illness uncertainty in caregivers of patients with neurological impairment, and low to medium levels were found in a study in pediatric patients and their caregivers^20^. In contrast with other studies, the data in this study show a first important finding, that is, the level of illness uncertainty experienced by the family caregiver is higher in the context of palliative care. Based on this finding, it can be inferred that this level is higher in the context of PC due to the complexity of the situation in palliative care. For instance, other studies show not only high levels of uncertainty, but also the presence of other phenomena that aggravate the situation at the end of life, such as stress, especially the spiritual and psychological stress. This is due to aspects such as responsibility overload in patient care, financial difficulties, self-abandonment and lack of free time^21^.

The correlations found in this study support the previous propositions, as according to the results, there is a significant association between the level of uncertainty in caregivers about the patient’s illness and the functionality and the symptoms of the patient. In this case, the level of uncertainty experienced by the family caregiver is likely to increase as the patient approaches the end of life and loses functionality or experiences more intense symptoms. Corroborating this hypothesis, the findings of a study showed that a higher level of illness uncertainty was associated with a lower functionality status in patients with stroke^18^.

Regarding the other characteristics in the caregiver profile, this study found a positive association between the level of uncertainty and the length of service as a caregiver, this finding is interesting compared to the results of a study in caregivers of patients with bowel cancer, which found that the level of uncertainty remained stable during one-year follow-up^22^. Regarding this finding, some studies^23^ have shown that the caregivers of a patient in PC learn about the disease and the care of their patient by a trial and error method, a situation that over time can contribute to acquisition of skills and experience, at least in the instrumental aspects of patient care.

Regarding the support provided to the family caregiver, a negative association was found between the uncertainty and the level of support perceived from health professionals. That is, the family caregivers have less uncertainty when they perceive more support coming from health professionals. This is a novel finding in the field of PC and supports one of the postulates of the Theory of Uncertainty in Illness, which states that authorities with credibility, in this case the health professionals, interfere in the emergence of illness uncertainty. This postulate had already been confirmed in other populations^24^but not in the context of PC. For instance, several authors^25-26^have shown that talking to health professionals about issues related to the patient’s situation is considered very important by caregivers and improves their knowledge about the management of the disease, empowerment in relation to their role, and decreases the uncertainty experienced.

Finally, the findings of this study report the importance of religious or spiritual environments and family support at the end of life. In this sense, a negative association was found between uncertainty and family support and religious support. As other authors have described^27-28^, family and spirituality are sources of support in the process of coping with the disease and provide accompaniment, help, financial support, tranquility to the caregiver and the patient, as well as sense and meaning at the end of life. This result confirms another assumption of the theory of Uncertainty in Illness in the field of palliative care, in which the author of this theory proposes that the provided support social is an important aspect that influences the development of illness uncertainty.

The implications that the results of this study have for nursing professionals are related to the importance of the inclusion of family caregivers as active subjects in the care for the patient under palliative care, since as this study shows, they have high levels of uncertainty about their relative’s illness. Secondly, the nursing professionals must bear in mind that, in the assessment and intervention of the family caregiver, various aspects may cause uncertainty, such as sociodemographic characteristics, the support network, the patient’s health status, the relationship of the caregiver with the health team, among others. Thirdly, both the nursing professional and the other members of the PC team must inform the caregiver in a clear and simple way about the patient’s illness, its progression and symptom management. In addition, they must support, guide and accompany the caregiver in coping at the end of the patient’s life and assist in accessing the available resources to manage the uncertainty that the illness of a patient in PC brings.

Finally, this study contributes to the advancement of knowledge in nursing, since the results show that the level of illness uncertainty in caregivers of patients in PC is higher when compared with other populations of caregivers. In addition, the findings of this study show an association between the level of uncertainty with the caregiver’s profile, specifically with the health status and symptoms of the patient in PC, the length of service as a caregiver and the perceived support from health professionals, family and religion. In addition, these associations prove that some of the postulates of the Theory of Illness Uncertainty apply to the field of the PC, and therefore, this research shows that this theory can be used in this field of care. On the other hand, the ability to develop further studies on the uncertainty in caregivers about the patient’s illness, including other variables related to both the practical experience and the results of the theory, would ultimately enable the development of more specific and detailed interventions in this context.

One of the limitations of this study can be related to the size of the associations found. In this regard, it is important to clarify that the uncertainty in caregivers about the patient’s illness is a complex and multidimensional phenomenon. For this reason, in many studies, although the associations seem weak, they are important and should be carefully observed since the issues addressed stems from various variables, which sometimes can not be measured in their entirety in the participants^29^. Another weakness of the study is related to the selection of the study participants, since an intentional sampling method was used, which did not allow a random assignment of the characteristics of the participants and this could introduce biases in the study results.

## Conclusion

Caring for a patient in palliative care is a complex situation that poses a challenge for the family caregiver. The results of this study show high levels of uncertainty in illness in family caregivers of patients in PC. In addition, these levels of uncertainty are associated, in a slight but significant way, with the condition of the patient who is cared for and the symptoms presented by him, the length of service as a caregiver and the support that the caregiver perceives coming from health professionals, family and religion. These findings provide evidence on the importance of the nursing professional in identifying the needs and assisting the family caregiver of the patient in PC; the strengthening of the family support network of this patient-caregiver dyad; and the need to recognize the value of religious or spiritual support groups. All this to avoid the appearance or to modulate the uncertainty in caregivers about the patient’s illness and to improve the health care provided to this population.
